# A Case of Mixed pitNET-Gangliocytoma of the Sella Turcica: Histopathologist’s take on Extremely Rare Occurrence

**DOI:** 10.12669/pjms.41.13(PINS-NNOS).13491

**Published:** 2025-12

**Authors:** Hasan Saeed, Madiha Khaliq, Mariam Abid, Zafar Ali, Amna Babar

**Affiliations:** 1 Hasan Saeed, MBBS, Shifa International Hospital, Islamabad, Pakistan; 2 Madiha Khaliq, MBBS, Shifa International Hospital, Islamabad, Pakistan; 3 Mariam Abid, MBBS, FCPS (Histopathology), FRCPath (UK) , Shifa International Hospital, Islamabad, Pakistan; 4 Zafar Ali, MBBS, FCPS (Histopathology), FRCPath (UK) , Shifa International Hospital, Islamabad, Pakistan; 5 Amna Babar, MBBS, FCPS (Histopathology), FRCPath (UK) , Shifa International Hospital, Islamabad, Pakistan

**Keywords:** Pituitary neoplasms, Neoplasm, Brain, Pakistan, Gangliocytoma

## Abstract

Mixed pituitary neuroendocrine tumors (pitNET)-gangliocytomas are extremely rare sellar region World Health Organisation (WHO) Grade-I tumors. We report the case of a 31-year-old Pakistani male with a presenting complaint of headache and gradual loss of vision for a period of few months. On magnetic resonance imaging (MRI), a sellar/ suprasellar and parasellar mass lesion was identified. A differential of pituitary macroadenoma was considered. The mass was excised and the surgery was uneventful. Histopathology revealed a biphasic tumor composed of small monomorphic nests and solid sheets of pitNET cells (synaptophysin and chromogranin A positive, S100 negative) and large voluminous eosinophilic cells of the gangliocytoma component (synaptophysin, chromogranin A and S100 positive). This case highlights a tumor with features of both neuronal and endocrine neoplasms. Although their presentation resembles that of pitNETs, their treatment response and behavior are more consistent with glioneuronal tumors, resulting in a highly favorable phenotype and treatment response.

## INTRODUCTION

Isolated gangliocytomas are extremely rare and mostly coexist with pituitary neuroendocrine tumors (pitNETs).^1^ Mixed pitNET-gangliocytomas, known to present with no specific clinical, endocrinological or radiological findings, account for 0.14-0.52% of all sellar tumors.^2^ More common in adult females, these are classified as neuronal and paraneuronal tumors according to the latest World Health Organisation (WHO) Neuroendocrine Tumor Classification Guidelines.^3^ The most common location is sella turcica alone or the sella turcica with suprasellar extension presenting most commonly with headache, acromegaly and visual disturbances. Endoscopic or microscopic transsphenoidal surgery is the main treatment modality utilized for tumor excision.^4^ Due to the similarity between the clinical and radiological presentations of these neoplasms, the final diagnosis is primarily established by histopathology.

Microscopically, two distinct neoplastic cell populations are observed in the same tumor mass with relatively clear separation between certain areas, the background fibrous tumor tissue predominantly consists of pitNET cells and the nodule portion primarily of ganglion cells.^5^ On immunohistochemistry (IHC), the ganglion cells are positive for synaptophysin and neurofilament protein (NFP), but negative for glial fibrillary acid protein (GFAP) while the adenomatous (pitNET) cells express synaptophysin, but not NFP. The Ki-67 labeling index is less than 5%.^6^

Here, we report a case of the coexistence of gangliocytoma and pitNET. To our knowledge, no prior case of mixed pitNET-gangliocytoma has been reported from Pakistan, making this the first documented case from the country. The histopathological details and expression of various IHC markers is highlighted, being the main diagnostic modality of this neoplasm.

## CASE PRESENTATION

A 31-year-old male presented in a Pakistani tertiary care hospital with the complaint of headache and decrease in vision for three months. Headache was gradual in onset and relieved with medications. On examination, patient had bitemporal hemianopia and bilateral pale disc on fundoscopy. There were no endocrinological signs of acromegaly or hyperprolactinemia. On magnetic resonance imaging (MRI), a sellar/ supra sellar and parasellar mass with hypointensity on sagittal T1-weighted imaging (T1WI) and hyperintensity on axial T2-weighted imaging (T2WI) measuring 2.2 x 1.8 x 0.7 cm was identified. A differential of pituitary macroadenoma was considered. Endoscopic transphenoidal surgery was performed and the tumor was excised. The surgery was uneventful. Preoperative serum growth hormone (GH) and adrenocorticotropic hormone (ACTH) levels were not assessed at the operating facility.

The surgical specimen was subsequently referred to Shifa International Hospital, Islamabad for histopathological evaluation analysis in October, 2024. Grossly, the tumor was fragmented, and collectively measured 1.5 x 1.0 x 0.5 cm. On hematoxylin and eosin (H&E) staining, a biphasic tumor was identified. The first component comprised of sheets and nests of small monomorphic cells having ovoid nuclei with finely stippled chromatin and granular eosinophilic cytoplasm. No mitoses were identified. The second component was composed of large ovoid to round ganglion-like cells scattered in a neuropil background.

**Fig.1A:**
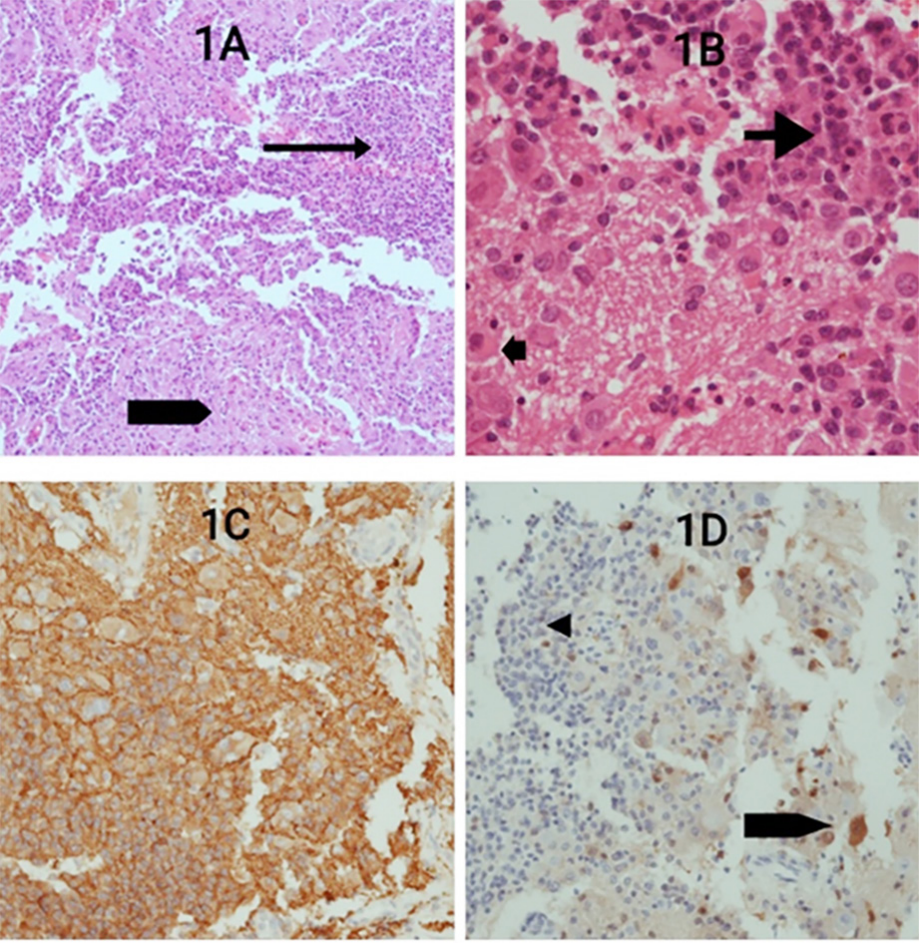
Photomicrograph showing a biphasic tumor with two cell populations; sheets and clusters of small monotonous cells in the top right (thin arrow) and large scattered cells in the bottom (thick arrow)(H&E,10X magnification); 1B) Photomicrograph showing the pituitary neuroendocrine tumor (pitNET) component comprising of round to oval cells with round nuclei and eosinophilic cytoplasm (long arrow), and gangliocytoma component comprising of large ganglion-like cells with cytoplasmic ballooning, occasional binucleation and eosinophilic granular bodies in a neuropil background(small arrow) (H&E,40X magnification); 1C) Synaptophysin immunostain: diffuse and strong cytoplasmic staining in both pitNET and gangliocytoma components; 1D) S100 immunostain: occasional nuclear and cytoplasmic positivity in the gangliocytoma component (arrow), and negativity in the pitNET component (arrow head).

The cells had voluminous cytoplasm with eosinophilic granular bodies and large centrally placed nuclei with in Conspicuous nucleoli. On IHC, both components were positive for neuroendocrine markers i.e. synaptophysin and chromogranin A and were negative for GFAP. Patchy nuclear and cytoplasmic positivity for S100 protein was identified only in the ganglion cell-like component. Proliferation index (Ki-67) was less than 4% (low). Neurofilament (NF) immunostaining, which is considered highly useful for distinguishing the gangliocytoma component from the pitNET component, was not performed due to non-availability of the antibody at our center (Fig.1).

On special staining (not shown), the reticulin meshwork was disrupted, excluding pituitary hyperplasia. Hence, a diagnosis of mixed pitNET-gangliocytoma was made. On follow-up approximately two months after surgery, the patient was stable with no evidence of tumor recurrence. The previously documented bitemporal hemianopia persisted without improvement and there was no further deterioration in vision.

## DISCUSSION

Isolated ganglion cell neoplasms (gangliogliomas and gangliocytomas) in the region of the sella turcica are extremely rare, mostly associated with a functioning/non-functioning pitNET. To date, fewer than 40 cases of mixed pitNET-gangliocytoma have been described in the literature, with some reports indicating up to one hundred cumulative cases globally. Despite having a benign clinical course, the associated pitNET component can produce GH and/or ACTH producing a clinical picture of acromegaly and/or Cushing’s disease respectively.^7^ Unlike this case, in which visual disturbances and headache were the only presenting complaints.

Three theories have been proposed explaining the histiogenesis of a mixed pitNET-gangliocytoma: (1) hypothalamic-releasing hormones produced by gangliocytoma may stimulate the adenomatous transformation of the pituitary gland; (2) gangliocytoma component may arise due to neuronal transdifferentiation of pitNET cells; (3) both gangliocytic and pitNET elements may originate from the pituitary pluripotent stem cells with bidirectional differentiation potentials.^4^,^8^ Shepard et al. reported a mean age of 44 years (range 28-63) and a female preponderance in a retrospective analysis of 10 mixed pitNET-gangliocytoma cases.^9^ Similarly, our patient lies in the same age bracket but is a male. In a study by Yang et al., on imaging the neoplasm was most commonly located in the intrasellar region, with suprasellar involvement and cavernous sinus involvement showing hypointensity on T1WI and hyperintensity on T2WI.^4^ Our case presented with a similar location and radiological characteristics without cavernous sinus involvement. Shepard et al. also reported a mean tumor diameter of 1.6 cm (range 0.4-2.4 cm) for which an endonasal submucosal microscopic transphenoidal surgery was performed comparable to our case.^9^

On histopathology, in a case study by Matyja et al. both of the neoplastic components were closely intermingled. The gangliocytic part showed clusters of large neuronal cells with occasional bi-nucleation and multinucleation scattered in a fibrillary background while the pitNET component was composed of clusters of small cells.^7^ These findings are in close accordance with our case. Another case showed the gangliocytoma component cells displaying Conspicuous nucleoli and abundant cytoplasm, with aggregated Nissl granules at the nuclear periphery. Similar to our case, where eosinophilic granular bodies were evident on microscopy.^7^

The evidence of dysplasia in any of the components of mixed pit-NET-gangliocytoma is rare. However, an extremely rare occurrence of a dysplastic cerebellar gangliocytoma (Lhermitte-Duclos disease) has been reported characterized by a germline loss of PTEN allele like Cowden disease.^10^ This highlights that although uncommon, gangliocytomas in different anatomical locations can occasionally display dysplastic changes and harbor identifiable genetic alterations, signifying the theoretical possibility of similar changes occurring in the neuronal component of sellar mixed tumors, although none were observed in our case.

Both the components of the neoplasm strongly and diffusely express neuroendocrine IHC markers like synaptophysim and chromogranin A. A recently reported case by Obiedat et al. described a mixed gangliocytoma-pituitary adenoma of dual lineage, emphasizing the importance of comprehensive immunohistochemical profiling, including transcription factor expression, to fully characterize such tumors.^11^ In a comprehensive systematic review by Balasubramanian et al., frequently positive IHC stains were growth hormone (GH) (78%), prolactin (59%), followed by synaptophysin (38%). GH and prolactin staining were not performed in our case due to lack of availability, although both components were diffusely positive for synaptophysin.^6^ Out of 17 cases, 59% had a proliferation index (Ki-67) of less than 3% highlighting the benign and slow growing nature of the neoplasm.^6^ Our case showed a low proliferation index of 4%.

Following surgery, the prognosis is generally good without major complications. Cossu et al. explained a general improvement of pre-operative symptoms especially visual disturbances following surgery. Rare recurrences, especially in cases of large tumors and iatrogenic pituitary insufficiency have also been reported.^1^

Sella turcica is an uncommon location for rare tumors like gangliocytomas. In addition to their occurrence with pitNETs, gangliocytic components have been described in association with other pathologies. For example, Zou et al. (2023) reported a unique case of IgG4-associated hypophysitis coexisting with MALT lymphoma and gangliocytoma, underscoring the diverse pathological contexts in which gangliocytic elements may be encountered.^12^ In case of a strong clinical suspicion of pitNET, complete excision, adequate sampling and comprehensive histopathological analysis should be executed to rule out the presence of a mixed gangliocytoma component. Since, these tumors are most commonly associated with GH and ACTH excess, complete hormonal profile and clinical examination must be performed and post-operative hormone levels should be assessed at regular intervals to check for hypopituitarism.

## CONCLUSION

A gangliocytoma co-existent with a pitNET is an uncommon diagnosis. Features of pituitary hormone excess are usually associated with this neoplasm akin to conventional pitNET. Hence, formulating a combined biochemical/radiology-based diagnosis is unreliable. Histopathology of the excised tumor with supporting immunohistochemistry is considered the gold standard for diagnosis. Despite having a favorable prognosis, adequate sampling and examination of a sellar mass lesion or a clinical pitNET is essential to identify any co-existent gangliocytoma component that might be overlooked on imaging or clinical examination.

### Authors Contribution:

**HS:** Conception, design, acquisition, analysis and interpretation of data, Drafted the manuscript.

**MK & MA:** Drafted the manuscript, analysis and interpretation of data.

**ZA:** Supervised, Critically reviewed the manuscript,Made final approval of the draft.

**AB:** Drafted the manuscript, analysis and interpretation of data.

All authors have approved the final version of the manuscript.
